# Effect of modified atmosphere package on attributes of sweet bamboo shoots after harvest

**DOI:** 10.3389/fpls.2024.1431097

**Published:** 2024-06-13

**Authors:** Ling Wang, Miyang Liu, Zheng Luo, Yulong Chen, Yingwei Qi, Mingqiang Ye, Feiping Chen, Fanwei Dai

**Affiliations:** Sericultural & Agri-Food Research Institute Guangdong Academy of Agricultural Sciences, Key Laboratory of Functional Foods, Ministry of Agriculture and Rural Affairs, Guangdong Key Laboratory of Agricultural Products Processing, Guangzhou, China

**Keywords:** bamboo shoots, postharvest changes, passive atmosphere modified package, lignification, cell wall components

## Abstract

Tender bamboo shoots undergo rapid senescence that influences their quality and commercial value after harvest. In this study, the tender sweet bamboo shoots (‘Wensun’) were packed by a passive modified atmosphere packaging (PMAP) to inhibit the senescence process, taking polyethylene package as control. The increase in CO_2_ and the decrease in O_2_ gas concentrations in the headspace atmosphere of the packages were remarkably modified by PMAP treatments. The modified gas atmosphere packaging inhibited the changes in firmness, as well as the content of cellulose, total pectin, and lignin in the cell walls of bamboo shoots. The enzymatic activities of cellulase, pectinase, and polygalacturonase that act on cell wall polysaccharides, and phenylalanine ammonia lyase, cinnamyl alcohol dehydrogenase, peroxidase, and laccase regulating the lignin biosynthesis were modified by PMAP treatment different from control during storage. The expression levels of the lignin biosynthesis genes *PePAL3/4*, *PeCAD*, *Pe4CL5, PeC4H, PeCCOAOMT, PeCOMT*, cellulose synthase *PeCESA1*, and related transcription factors *PeSND2, PeKNAT7, PeMYB20, PeMYB63*, and *PeMYB85* were clearly regulated. These results suggest that PMAP efficiently retards the changes in lignin and cell wall polysaccharides, thus delaying the senescence of tender sweet bamboo shoots during storage.

## 1 Introduction

As one of the most natural and healthy stem-edible vegetables, bamboo shoots, belonging to the *Gramineae* family, are widely consumed in China and other Southern Asian regions (Zeng et al., 2015). The tender bamboo shoots contain a variety of active ingredients, such as carbohydrates, proteins, minerals, vitamins, dietary fibers, and antioxidants (Zhang et al., 2024). However, post-harvest bamboo shoots are still living organisms with vigorous respiration and physiological activities that induce a rapid decline in bamboo shoot quality (Shi et al., 2023). It is known that the texture change in bamboo shoots is attributed to unusual alterations in cell wall metabolites, particularly those in the stem lignification process (Zheng et al., 2020a). Lignification in the stem of bamboo shoots is accompanied by an increase in cellulose and lignin content, inducing textural changes with an increase in firmness, a decrease in water content and nutrients, and a bright yellow or dull appearance (Zheng et al., 2020b; Liu et al., 2023a).

Numerous postharvest applications such as nitric oxide, brassinolide, UV light radiation, and melatonin to alleviate lignification changes in bamboo shoots have been evaluated (Qi et al., 2020; Zheng et al., 2020a; Liu et al., 2023b; Yang et al., 2023). These treatments exhibited the capacity to inhibit the reactive oxygen signaling changes, and the activities of phenylalanine ammonia-lyase (PAL), peroxidase (POD), and cinnamyl alcohol dehydrogenase (CAD) after harvest (Qian et al., 2023). The biosynthesis and polymerization of lignin monomers involve a series of enzymes as well as their corresponding genes derived from the phenylpropanoid pathway (Zhao, 2016). In addition, numerous transcription factors in the NAC and MYB families are involved in regulating lignification in post-harvest bamboo shoots (Zhong and Ye, 2015; Zhang et al., 2018). For instance, the relative expression levels of MYB20, MYB85, SND2, and KNAT7 were not regulated by the lignification process but were suppressed by postharvest treatments in bamboo shoots during storage (Li et al., 2019). Therefore, appropriate strategies to inhibit the increase in enzyme activity, rapid oxidation process, and changes in the expression levels of regulatory genes are important to control the rapid lignification of bamboo shoots during storage.

Modified atmosphere packaging has been used in food preservation to regulate quality and extend shelf life (Qu et al., 2022). Passive modified atmosphere packaging (PMAP) can automatically regulate the gas concentrations of O_2_ and CO_2_ in the package, which can inhibit oxidation reactions, microbial spoilage, and shrinkage and maintain quality (Sandhya, 2010). PMAP applies different packaging films with various permeabilities for gas and water to modify atmospheric O_2_, CO_2_, and relative humidity (RH), thereby prolonging the storage of fruits and vegetables (Yang et al., 2018). Song et al. indicated that the application of PMAP and low-temperature storage alleviated the lignification process and maintained membrane integrity, thereby extending the shelf life of sweet bamboo shoots (Song et al., 2013). These studies commonly used polymeric films that have various permeabilities for O_2_ and CO_2_ in a packed atmosphere, thus extending the shelf life of fruits and vegetables (Cantín et al., 2008). However, it is important to select the appropriate size and permeability for different fruits and vegetables.

The present study was set to evaluate the application of a suitable PMAP to prolong the shelf life of bamboo shoots during storage. Multiple physiological and chemical analyses of quality changes, especially the lignification process of postharvest bamboo shoots, were comprehensively addressed. The effects of PMAP treatment on atmospheric gas concentration, texture firmness, cellulose, total pectin, and lignin content in sweet bamboo shoots were assayed. Key enzymes involved in lignin biosynthesis and changes in cell wall polysaccharides, as well as their corresponding gene profiles, were analyzed. Furthermore, the potential regulation of PMAP at the physiological and biochemical levels to delay the senescence of bamboo shoots after harvesting is discussed.

## 2 Materials and methods

### 2.1 Materials and treatments

The sweet bamboo shoots (*Phyllostachys elegans* McClure) as ‘Wensun’, which is widely planted in south China as a traditional forest vegetable, is selected to investigate the postharvest storage changes. The tender bamboo shoots of *P. elegans* were picked up from a farmland plantation field in Guangning, Guangdong Province of China (112°03′~112°43, 22°22′~23°59′). Tender bamboo shoots with fine coatings were immediately transported back to lab, and cooled down for 12 h at 8°C. The leaf sheaths that cover on the outer surface of bamboo shoots were carefully peeled off. Subsequent peeled bamboo shoots with no obvious damages and uniformity of size, were selected for the preservation experiments. Prior to the treatments, the selected bamboo shoots were dipped in 0.02% (w/v) sodium hypochlorite for 30 s, and air dried. Then, the samples were divided randomly as two groups, respectively. One group of samples were placed in PMAP (MP40, bag thickness of 40 μm; the size of package was 25cm×35cm, O_2_ permeability of 4632 cm^3^ m^−2^ d^−1^; CO_2_ permeability of 2316.5 cm^3^/m^2^/day/atm). The passive with different thickness of 20 µm (MP20), 30 µm (MP30), and 40 µm (MP40) were tested, and found that MP40 exhibited better efficiency than those other treatments. Therefore, we selected MP40 as the formal treatment for this presented study. The samples in one group were packed with conventional commercial polyethylene packages (O_2_ permeability of 20083 cm^3^ m^−2^ d^−1^) and set as the control. The samples of 10 bamboo shoots were packed in each bag, 3 parallel packages per treatment for each storage stage were prepared, and all the samples were stored under 4°C. During storage, samples at 0, 4, 8, 12 and 16 d, and the packed atmosphere gas concentration, firmness of sweet bamboo shoots were assayed and the samples were crashed and stored at -80°C for further analysis.

### 2.2 Assay of gas concentration in the modified packages and shoot firmness

The gas concentration mainly containing O_2_ and CO_2_ for the headspace of the packages were analyzed according to our previous method (Wang et al., 2020a). The gas concentration inside the bags were measured with four different positions in the package (top, down, front, and back) with the gas analyzer (PBI Dansensor, Ringsted, Denmark). As mentioned above, the headspace gas in three packages at each stage for each treatment were analyzed. The gas concentration was indicated using the % of overall gas in the packages.

The changes in firmness of the bamboo shoots were measured using a digital force pressure, which was equipped with a probe with diameter of 2 mm. The bamboo shoot samples at each storage were cut into different segments with 30 mm length for the firmness assay. The segments were penetrated at 10 mm with a speed of 1.0 mm s^−1^ and the maximum force was recorded (Textural apparatus, Leqing, China). Ten bamboo shoots from each bag of different treatments at each time point were determined. The firmness for the bamboo shoot was expressed as N.

### 2.3 Determination the content of the lignin, total pectin, cellulose

The main cell wall content of the bamboo shoot was assayed using the previous reported method with minor modifications (Li et al., 2019). The frozen bamboo shoot samples (3.0 g) were grinded in powder and immersed in 95% ethanol about 30 mL for three times, and each time was centrifuged at *×* 5000 *g* for 10 min at 4°C. For the lignin analysis, acetyl bromide-glacial acetic acid with 2 mL at 25% (v/v) were added into the collected pellets, and the mixture solution was with water bath for 30 min under 80°C. After that, 1 mL of 1 M NaOH was added into the mixtures to terminate the reaction. The solution was further mixed with glacial acetic acid (1 mL) and hydroxylamine hydrochloride (0.1 mL, 7.5 M). Then, the absorbance values of mixtures were recorded under 280 nm.

For the overall pectin content determination, the residues were hydrolyzed with diluted acid to get soluble pectin, which could convert into galacturonic acid. The products were condensed with carbazole. Then, the absorbance values of the extracts were recorded under 530 nm.

For the measurement of cellulose, the residues were mixed with nitric acid-ethanol solution and incubated under 100°C for 1 h, the celluloses were decomposed into β-D-glucose. Then, the mixtures were measured the absorbance 620 nm. The content of lignin, total pectin, and cellulose were expressed as mg g^−1^ based on fresh weight.

### 2.4 Enzyme activity analysis

The changes in activity for the enzymes in lignin biosynthesis and cell wall polysaccharides modifications were analyzed to indicate their activities during senescence in bamboo shoots. Briefly, the frozen powder with 1 g of bamboo shoot tissues in each sample were homogenized with extraction buffer (5 mL), containing 100 mM sodium acetate, 0.2% Na_2_S_2_O_4_, and 1% PVP (w/v). Then, the homogenized mixtures were centrifuged at 12,000g, which was set under 4°C for 20 min. The supernatants containing crude enzymes were obtained, which was used for further enzyme activity assay. Activity of polygalacturonase (PG, EC 3.2.1.15), pectinase (EC 3.2.1.15), cellulase (EC 3.2.1.4), PAL (EC 4.3.1.24), POD (EC 1.11.1.7), laccase (EC 1.10.3.2), and CAD (EC 1.1.1.195), were analyzed according to reported methods with minor modifications (Wang et al., 2020a). The enzyme activity was recorded as U g^-1^ based on fresh weight.

### 2.5 Obtaining of RNA and relative expression analysis by qRT-PCR

To elucidate the changes in lignin biosynthesis and metabolism of cell wall polysacharrides on gene expression level, quantitative PCR for those key genes involved in these metabolisms were analyzed. Total RNA of bamboo shoots was extracted using RNA Extraction Kit (Tiangen, Beijing, China). The crude RNA was then purred using DNase I (TaKaRa, Otsu, Japan), and the concentration of RNA for different samples were evaluated by microplate detector (Spark^®^, Tecan, Shanghai, China). After the clean RNA was obtained, the cDNA synthesis was performed using the meScript^®^ RT reagent kit (Takara, Dalian, China). The mixture action of the PCR analysis for gene expression was tested with SYBR^®^ Premix Ex TaqTM II (TaKaRa, Dalian, China) on a CFX Connect Real-Time PCR System (Bio-Rad, California, USA). Relative expression levels of the selected genes involved in the lignin biosynthesis and cell wall polysaccharides (*PePAL*, *PeCAD*, *Pe4CL*, *PeC4H*, *PeCOMT*, *PePOD*, *PeCESA*, *PeKNAT*, and *PeMYB*) were analyzed. The gene of CAC as reference gene for the relative expression level calculation, was selected according to previous reported studies on bamboo shoots (Fan et al., 2013). The primer sequences for all the tested genes used in RT-qPCR analysis have been listed in **Table S1**. The relative expression levels were determined using the 2^-ΔΔCT^ method (Schmittgen and Livak, 2008).

### 2.6 Statistical analysis

All samples with three biological replicates were performed, and the results were presented as mean values with standard errors. The statistical analysis involved in multiple samples and comparison were tested using the one-way analysis of variance (ANOVA). The significance differences were carefully assayed, and the analyses were performed with significant level of 0.05. The graphs were performed using SigmaPlot.

## 3 Results and discussion

### 3.1 Changes in the appearance of bamboo shoots and gas concentration in the packages

**Figure 1:**
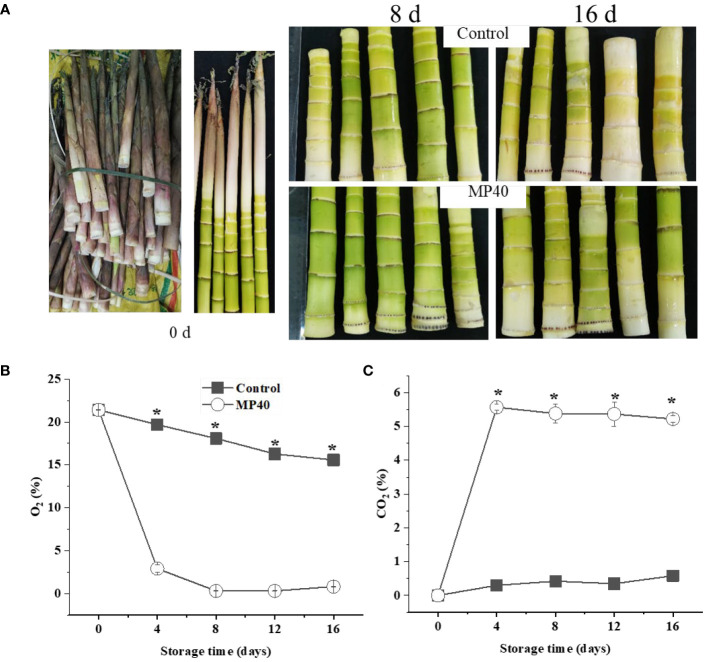
The overall visual changes of sweet bamboo shoot (*P. elegans*) after passive modified atmosphere packaging (MP40) treatments during storage. **(A)** The visual appearance of sweet bamboo shoot at 0 d, 8 d, and 16 d at 4°C after treatments during storage. Relative content of **(B)** O_2_ and **(C)** CO_2_ in the packed atmosphere. Data were represented as a mean ± standard errors (SE). Asterisks in graphs indicated the significantly different (*P* ≤ 0.05).

Edible sweet bamboo shoots at the harvest stage developed green tender stems, which were prone to senescence, and exhibited a yellow appearance and increased stem hardness during storage. As shown in **Figure 1A**, the tender sweet bamboo shoots developed a green color coated by the leaf sheath at the harvest stage, and the freshly cut samples changed to yellow, with partly green, which could be slowed down by the PMAP. PMAP treatment has been widely applied to modify atmospheric gas concentrations in packages, thus regulating quality changes in both fruits and vegetables (Wang et al., 2020a; Mou et al., 2023, Mou et al, 2023). During the entire storage period, the O_2_ concentration decreased slowly and remained at approximately 15% at the end of storage in the control, while the O_2_ level of PMAP samples was lower than that (*P*<0.01). The O_2_ content decreased significantly at 4 d (2.9%) and then changed slowly thereafter in the treated group (**Figure 1B**). The CO_2_ content increased dramatically at 4 d and then remained stable in MP40 but changed slowly and increased to 0.5% at 16 d in the control (**Figure 1C**). The atmospheric concentrations of O_2_ and CO_2_ in the package depend largely on the permeability of the packaging bags during storage. The effects of PMAP on the quality changes of different horticultural crops have been widely evaluated, such as Chinese flowering cabbage, mulberry leaves, and pea pods after harvest (Wang et al., 2020a; Yang et al., 2022; Mou et al., 2023, Mou et al, 2023; Luo et al., 2024). All the reported examples indicated that PMAP could efficiently regulated the gas concentrations inside of the packages, which might be induce series of physiological, biochemical, and molecular level regulations to delay the senescence process (Wang et al., 2020b; Mou et al., 2023). In the present study, the storage circumstance of O_2_ and CO_2_ concentrations was regulated by PMAP, which delayed the senescence of sweet bamboo shoots during storage.

### 3.2 Changes in sample firmness and the cell wall metabolites

**Figure 2:**
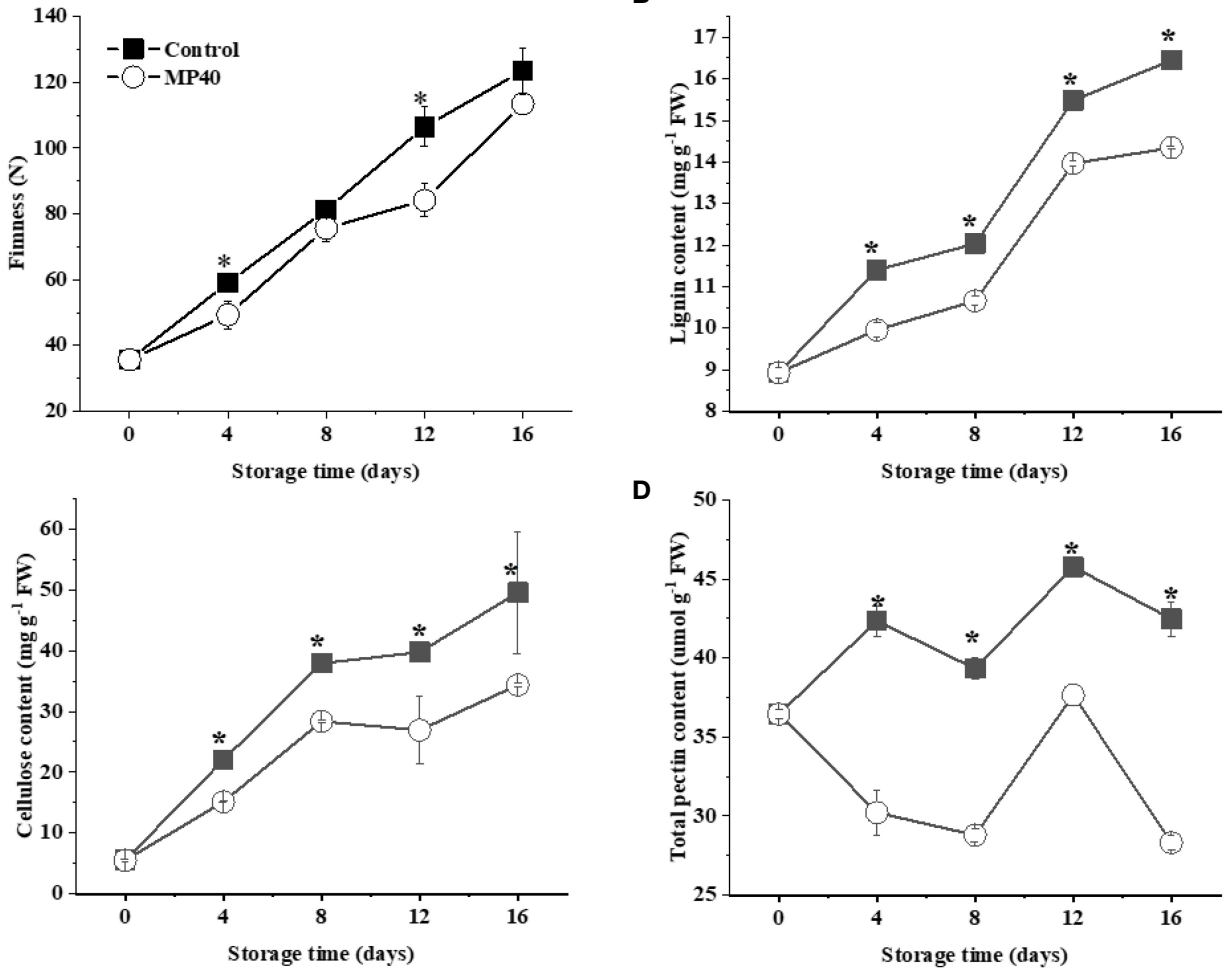
Changes in texture and main cell wall components in sweet bamboo shoot during storage. The changes in **(A)** firmness, **(B)** lignin content, **(C)** cellulose content, and **(D)** pectin in sweet bamboo shoot. Data were represented as a mean ± standard errors (SE) on basis of fresh weight. Asterisks in graphs indicated the significantly different (*P* ≤ 0.05).

Stem texture, including firmness and color, changes rapidly following the hardening and senescence of tender sweet bamboo shoots after harvest (Cesarino, 2021; Yang et al., 2023). The tender bamboo shoots had a firmness of around 35.6 N, and this increased gradually to 106 N at 12d, which could be apparently inhibited by MP40 (firmness of 84 N; **Figure 2A**). The firmness of the stems of horticultural crops is largely related to cell wall metabolites, such as pectin, cellulose, and lignin, etc (Wang et al., 2023; Yang et al., 2023). The growth of bamboo shoots is usually accompanied by an increase in thickness and related components that enhance the adhesion of cells through intercellular interactions and the mechanical strength of the cell wall (Cesarino, 2021). Appropriate treatments may be necessary to inhibit rapid alterations in cell wall components and lignification, thus maintaining the tender taste and quality of bamboo shoots after harvest.

The quality changes could be efficiently maintained by physical treatments with PMAP in both fruits and vegetables, such as Chinese flowering cabbage (Mou et al., 2023), bell pepper (Frans et al., 2021), and mulberry leaf vegetables (Yang et al., 2022). The treatment of PMAP was shown to modify the headspace gas concentration to further regulate oxidation and a series of physiological and biochemical senescence changes in the samples (Qu et al., 2022). In sweet bamboo shoots, the lignin content increased gradually with the extension of storage time, but it was slowed down by MP40 compared with the control (**Figure 2B**). As has been widely mentioned, the accumulation of lignin in plant tissues exhibited a directly effect on stem firmness in Chinese flowering cabbage and Moso bamboo shoots (Kamdee et al., 2014; Wang et al., 2020a; Wang et al., 2023). Therefore, the rapid increase of lignin content might be one of the main factors inducing the hardening changes in sweet bamboo shoots during storage (Yang et al., 2021). The mechanical properties of plant tissues are largely affected by the cell wall components including lignin and the primary cell wall polysaccharides (Liu et al., 2023a; Zhang et al., 2021a). In sweet bamboo shoots, the cellulose content increased rapidly, reaching double that of control samples at 16 d after harvest (**Figure 2C**). Total pectin content in BBS changed slowly until 12 d, then increased dramatically in control samples during storage (**Figure 2D**). Accordingly, the increase in firmness and cell wall components was inhibited by the MP40 treatment during storage. Tender bamboo shoots have increased lignin and cellulose content, inducing a short shelf-life (Zhang et al., 2021b), which could be alleviated by MP40, extending the shelf life after harvest.

### 3.3 Enzyme activities and the expression of metabolism-related genes for the cell wall polysaccharides and lignin

**Figure 3:**
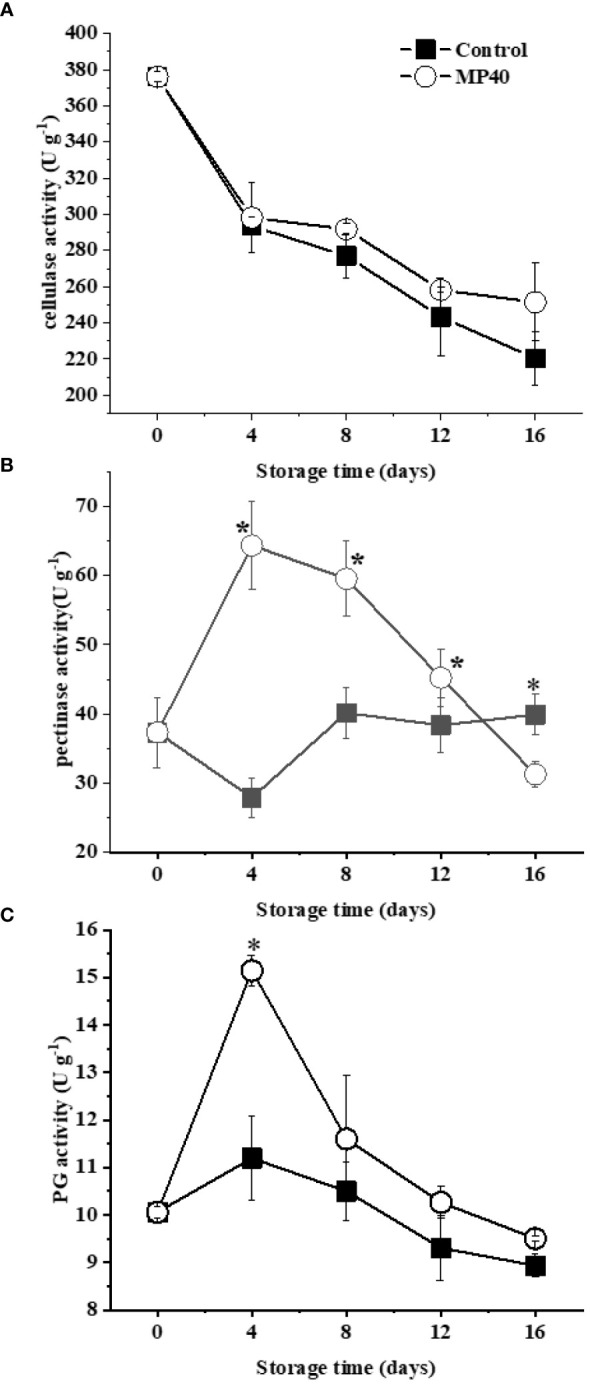
Activity of enzymes involved in changes of cell wall polysaccharides in sweet bamboo shoot during storage. Changes in activity of **(A)** pectinase, **(B)** cellulase, and **(C)** PG. Data were represented as a mean ± standard errors (SE) on basis of fresh weight. Asterisks in graphs indicated the significantly different (*P* ≤ 0.05).

As changes in cell wall constituents are largely affected by their related enzymes (Zhang et al., 2021a), the activities of enzymes involved in cell wall polysaccharide and lignin metabolism were assayed. During storage, cellulase activity in both the control and MP40 samples displayed a decreasing trend (**Figure 3A**). Pectinase activity increased gradually and peaked at day 4 but declined rapidly in the MP40 samples, whereas it changed slowly in the control (**Figure 3B**). PG (Polygalacturonase) activity exhibited an increasing trend and peaked on day 4, which was higher in the MP40 group than in the control ((**Figure 3C**). The prominent components of the primary cell wall are pectin, cellulose, and hemicellulose, which form the matrix for the mechanical properties (Wang et al., 2018). Changes in the activity of pectinase, cellulase, and PG may modify the degree of esterification in the pectin matrix, polysaccharides, and polygalacturonic acid chains of the cell wall (Gall et al., 2015; Wang et al., 2018). In addition, modification of the activity of these enzymes may affect cell–cell adhesion and mechanical strength (Zhang et al., 2020; Liu et al., 2023a). Fruit ripening and/or softening is largely attributed to the primary cell wall with progressive disassembly changes induced by a series of cell wall hydrolases (Brummell, 2006). Treatment with MP40 may cause changes in cell wall components, including both primary and secondary components, by modifying enzyme activities to delay storage time.

**Figure 4:**
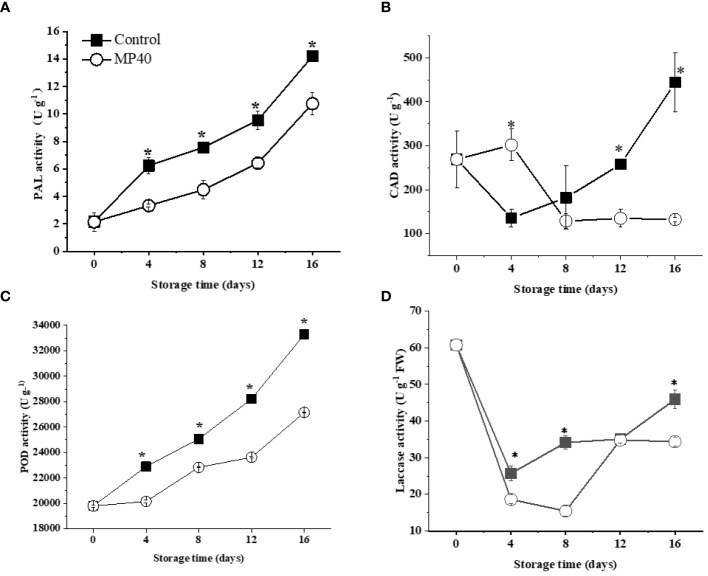
Activity of enzymes involved in lignin biosynthesis in sweet bamboo shoot during storage. Changes in activity of **(A)** PAL, **(B)** CAD, **(C)** POD, and **(D)** laccase. Data were represented as a mean ± standard errors (SE) on basis of fresh weight. Asterisks in graphs indicated the significantly different (*P* ≤ 0.05).

In contrast to the primary cell walls with polysaccharides, lignin is a series of phenylpropanoid derivatives that polymerize as a matrix in the secondary cell wall (Zhao, 2016). Consistent with the increase of lignin content, the activities of some key enzymes for lignin formation, such as PAL, CAD, POD, and laccase, exhibited increasing trends in sweet bamboo shoots after harvest (**Figure 4**). As shown in **Figures 4A, C**, the enzyme activities of PAL and POD displayed continuously increasing changes in both the control and MP40 samples, but exhibited lower levels in MP40 samples during storage. CAD activity increased at 4 d and then decreased in the MP40 group, while it decreased in the first 4 d and increased dramatically thereafter in the control samples (**Figure 4B**). Laccase is the key enzyme involved in the polymerization of lignin (Hoffmann et al., 2020); its activity decreased slightly at 4 d and then increased significantly, while it was lower in MP40 samples than in the control (**Figure 4D**). It has been suggested that lignification in bamboo shoots is a complex process involving several enzymes such as PAL, CAD, and POD (Luo et al., 2008). The findings of this study suggest that MP40 treatment can modulate the enzymatic activities of lignin metabolism enzymes, thereby reducing the accumulation of lignin in sweet bamboo shoots.

**Figure 5:**
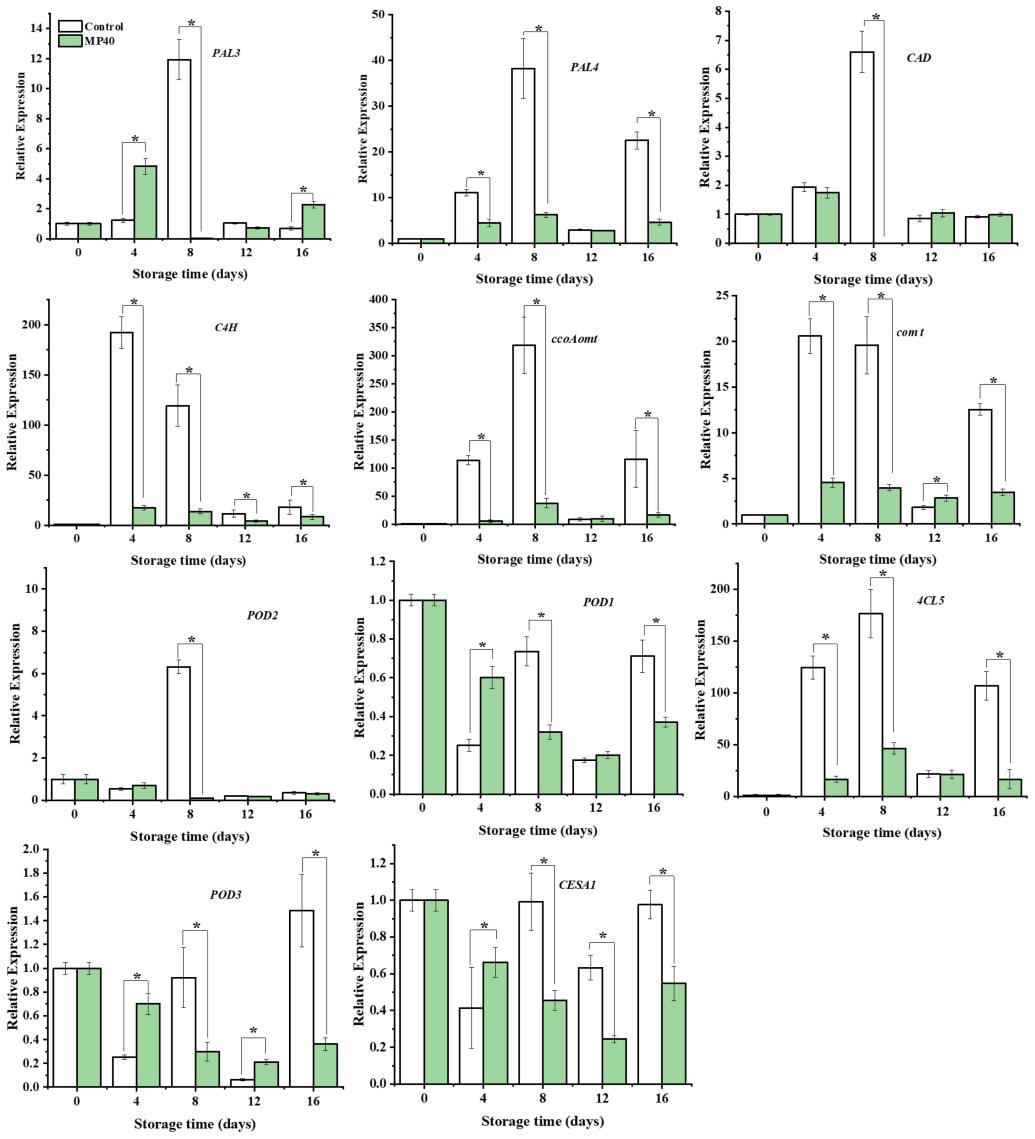
Relative expression of lignin biosynthesis and cell wall polysaccharides metabolism-related genes in sweet bamboo shoot during storage. Data were represented as a mean ± standard errors (SE). Asterisks in graphs indicated the significantly different (*P* ≤ 0.05).

**Figure 6:**
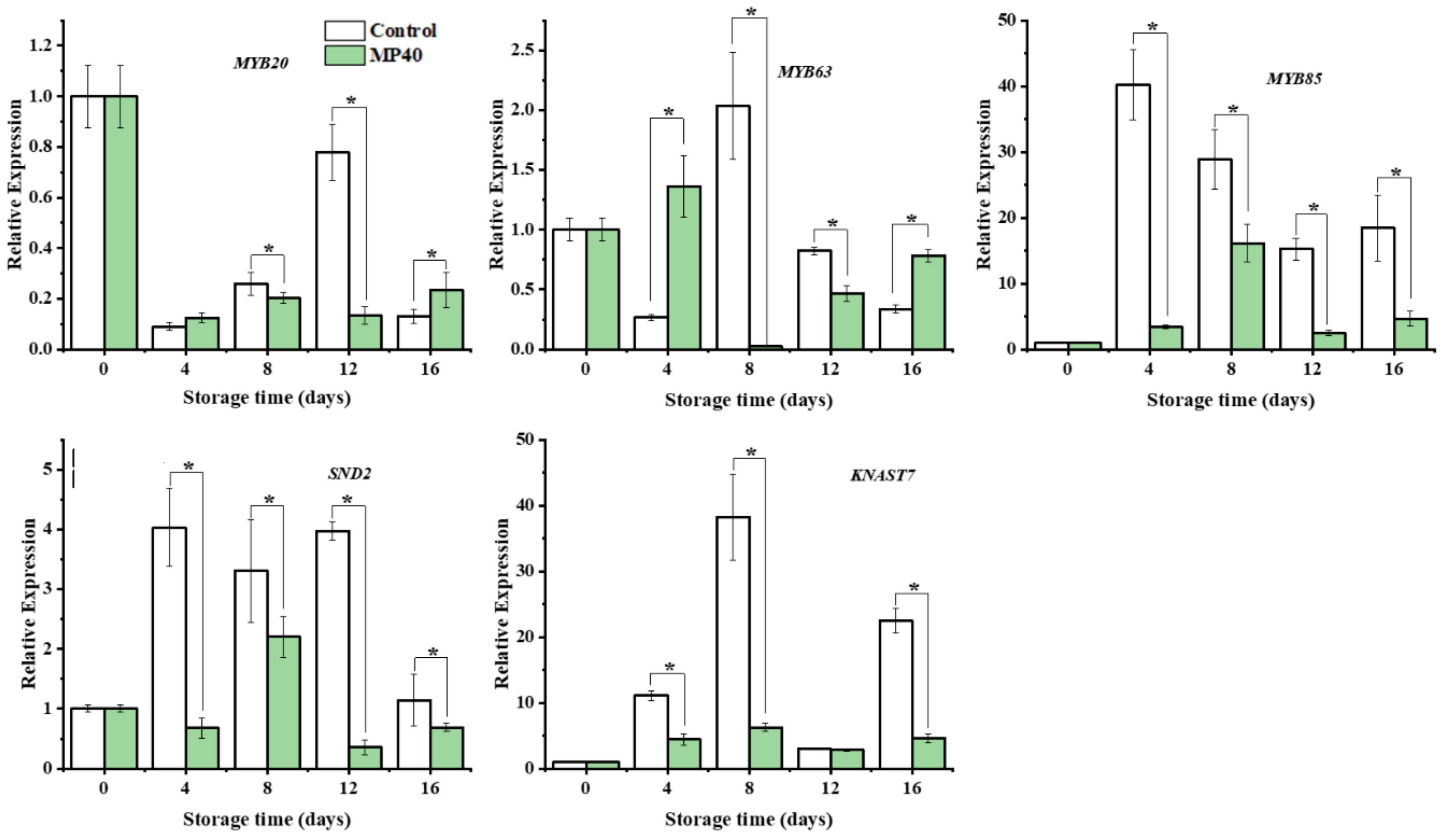
Relative expression of the transcription factors that involved in lignin biosynthesis regulation in bamboo shoots. Data were represented as a mean ± standard errors (SE). Asterisks in graphs indicated the significantly different (*P* ≤ 0.05).

Changes in enzyme activity and metabolite content are usually accompanied by dynamic changes in corresponding genes (Rao and Barros, 2023). The initially limited enzyme gene *PePAL3/4*, biosynthesis of lignin monomers and polymerization genes (*PeCAD*, *Pe4CL*, *Pe4CL5, PeC4H*, *PeCCOAOMTt*, *PeCOMT*, *PePOD1*, *PePOD2*, and *PePOD3*), and the cellulose synthase gene *PeCESA1* were overall downregulated, with lower expression levels in the MP40 samples than in the control (**Figure 5**). Cell wall metabolism, including primary cell-wall polysaccharides and secondary cell-wall components, is regulated by several transcription factors in plants. Among these transcription factors (TFs), the NAC and MYB superfamilies have been reported to play important roles in cell wall formation (Zhong et al., 2010). The relative expression of *PeSND2*, a transcription factor, increased dramatically at 12 d and then decreased rapidly at 16 d in the control. *PeMYB20/63* exhibited the highest expression level at 12 d and 8 d in the control. The expression of *PeMYB85* increased rapidly with peaking time at 4 d in the control, while all the evaluated TFs exhibited lower expression levels in the MP40 samples compared to the control group. The expression of *PeKNAT7* increased following the senescence of bamboo shoots in the control, and MP40 treatment inhibited its expression (**Figure 6**). The transcription factor KNAT7 is a negative regulator and a target of MYB46 and NST3/SND1, involved in the formation of secondary cell walls in plants (Ko et al., 2009; Wang et al., 2020b). The expression of these TFs was also inhibited by melatonin, which was used to slow postharvest senescence in Moso bamboo shoots (Li et al., 2019). The fluctuated changes of relative expression of these texted genes might be induced by the adaptation of senescence changes affected by the low temperature. As the occurrence of senescence changes, the expression of lignin-related genes increased, which might be induced by the reactive oxygen signal, and promoted the increase of lignin and hardening of sweet bamboo shoots after harvest (Yang et al., 2021; Qian et al., 2023). The treated samples packed with MP40 packages exhibited overall lower expression levels for those metabolism genes and transcriptional factors than control (**Figures 5**, **6**). These results suggest that MP40 treatment may influence transcriptional regulation to affect lignification and cell wall changes, thereby mediating the senescence of sweet bamboo shoots during storage.

**Figure 7:**
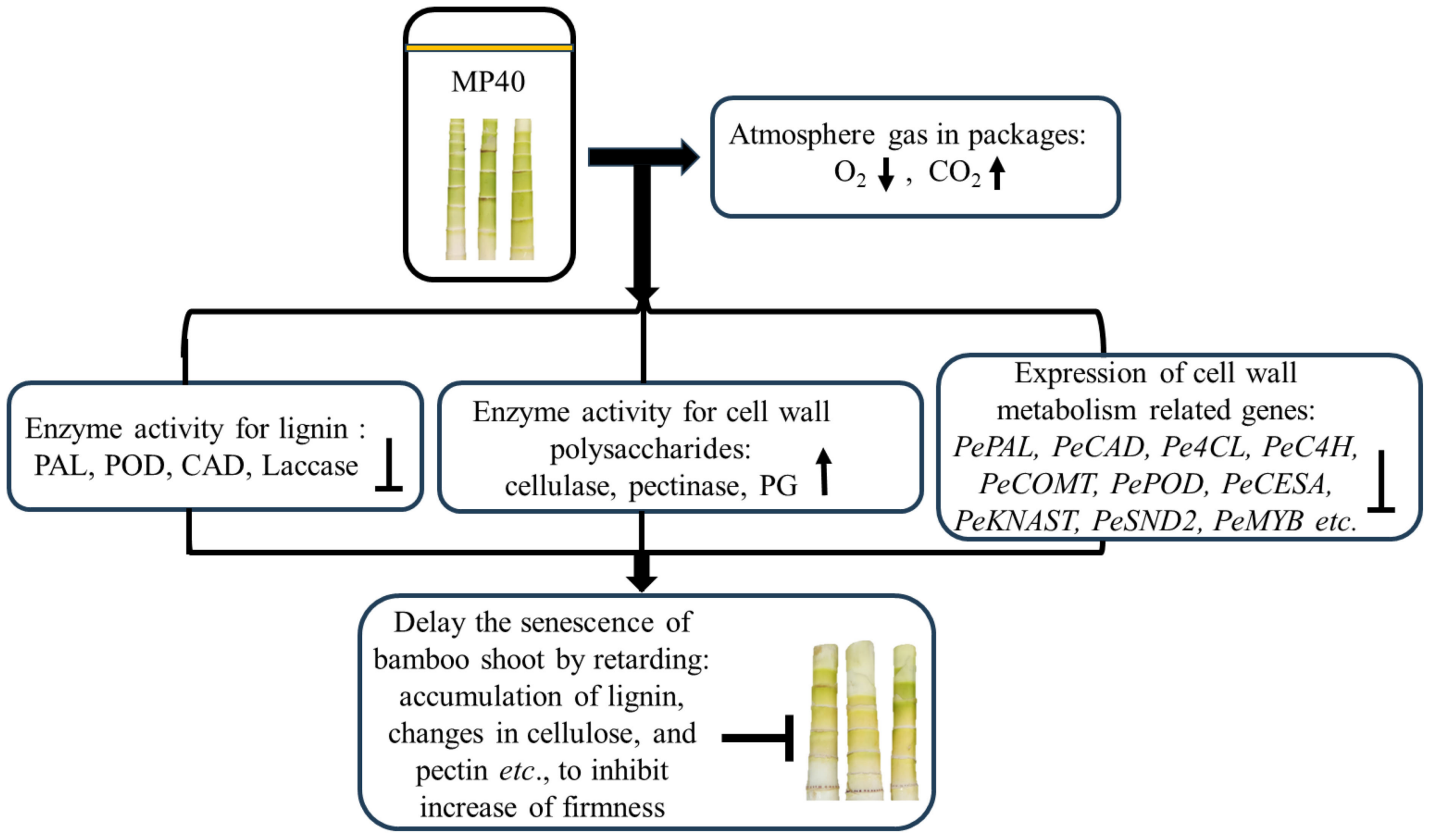
The overview of potential regulation process of passive modified atmosphere packaging to delay senescence of sweet bamboo shoot. The MP40 treatments may modify the atmosphere gas content to inhibit the rapid biosynthesis of lignin and changes in cell wall polysaccharides during storage.

As a physical treatment, PMAP efficiently regulates the headspace gas concentrations in packages, which can slow down the rapid ripening and/or senescence of both fruits and vegetables (Pinto et al., 2020; Wang et al., 2020a; Mou et al., 2023). Bamboo shoots accumulate abundant primary and secondary cell wall components, including cellulose, pectin, and lignin polymers, which enhance their mechanical strength during the growth period (Yang et al., 2021). In the present study, the application of MP40 automatically regulated the equilibrium of the atmosphere in the packaging and retarded the increase in firmness, lignin, and cell wall polysaccharides. Treatment with MP40 may modify the activity of enzymes involved in regulating both cell wall polysaccharides and lignin metabolism, and the relative expression of the corresponding genes for these enzymes was also altered to retard the rapid senescence of sweet bamboo shoots after harvest (**Figure 7**).

## 4 Conclusion

The present study investigated the effect of PMAP on senescence in tender sweet bamboo shoots in relation to retarded cell wall metabolism. The application of PMAP modified the headspace gas concentrations by promoting a rapid increase in CO_2_ and a decrease in O_2_ in the packages, thereby maintaining the equilibrium of gas composition in the packages. The firmness increased in tender bamboo shoots due to an increase in the cell wall polysaccharides cellulose, pectin, and lignin, which were considerably inhibited by PMAP. Simultaneously, the activities of enzymes involved in cell wall polysaccharide and lignin metabolism, and the corresponding genes and transcription regulators for primary and secondary cell wall formation were also significantly modified after the treatment. This further indicates that inhibition of lignin and cell wall component accumulation could affect changes in the hardness of bamboo shoots after harvest. These results suggest that PMAP enhances the capacity to retard changes in lignin and cell wall polysaccharides, thus delaying the senescence of bamboo shoots. This may also lay the foundation for the search for appropriate strategies to prolong the shelf life of tender bamboo shoots during storage. However, the molecular mechanisms involved in physiological and metabolic processes, such as energy status and ROS balancing system, in the context of storage, need further investigation. These results suggested that PMAP was successful and efficient in delaying quality changes and suitable for nutrition maintenance and lignification inhibition during the postharvest storage of bamboo shoots.

## Data availability statement

The raw data supporting the conclusions of this article will be made available by the authors, without undue reservation.

## Author contributions

LW: Conceptualization, Formal Analysis, Funding acquisition, Investigation, Writing – original draft, Writing – review & editing. ML: Formal Analysis, Writing – original draft. ZL: Formal Analysis, Investigation, Writing – original draft. YC: Formal Analysis, Investigation, Methodology, Writing – original draft. YQ: Formal Analysis, Investigation, Writing – original draft. MY: Formal Analysis, Writing – original draft. FC: Formal Analysis, Writing – original draft. FD: Conceptualization, Funding acquisition, Writing – original draft.
