# Supplementary material for: Effect of modified atmosphere package on attributes of sweet bamboo shoots after harvest
Source: Front Plant Sci. 2024 Jun 13;15:1431097. doi: 10.3389/fpls.2024.1431097 (PMC11212469; doi:10.3389/fpls.2024.1431097)
Supplement: Supplementary file 1 [file Table_1.pdf]

Table S1. List of primers used in quantitative real-time PCR.

Gene ID	Gene Name	Primer sequence (5'–3')(Sense/Anti-Sense)	References
PH01085796G0010	*PAL3*	CGAGGAGAACGTCAAAAGTGCC<br>CTTGAGCAGGTCCTTTTCGCAG	(Li et al., 2019)
PH01003309G0050	*PAL4*	GAGACCTCCATCTTCGCCAAGG<br>GTACGACCGGCACTCCTTGATC	(Li et al., 2019)
PH01006980G0020	*POD1*	GCGAGTGATGCTGCCTATGAAC<br>AAACCTGTCCACCCTTCAGTCC	(Li et al., 2019)
PH01000166G0130	*POD2*	GGACATCTTCTTCCAGCACTTCG<br>GTTGTTCCTGATCTCGCCGTTG	(Li et al., 2019)
PH01004068G0100	*POD3*	GACAACGCCTACTTCCACGACA<br>GACGAACCTGCAGTCCTTCCT	(Li et al., 2019)
PH01004840G0070	*CAD*	GCTGCTGAATGCGAGAATGG<br>TACTTTGTACAGCCCTGCGG	(Zhang et al., 2018)
PH01002285G0270	*C4H*	AACTACGGCGACTTCATCCC<br>TCACCTGTCTGTGCCATCAC	(Zhang et al., 2018)
PH01000664G0640	*CCoAOMT*	GCGCTTGAAGAGCTCAACTT<br>CCTCCAGTCCTTGACAGCTC	(Zhang et al., 2018)
PH01000201G0400	*CoMT*	TGCCATCACCATTCGAGGAC<br>GAGACTCCAGCCCCTTGAAC	(Zhang et al., 2018)
PH01001175G0060	*CESA1*	TTACCAATCTTGGGGTCCGC<br>TTGACGCAAGGAGGATGGAC	(Zhang et al., 2018)
/	*4CL5*	ATGTCGGGTACGTTGACGAT<br>TGATGTCATCTTCGCTGAGG	(Hou et al., 2022)
PH01002092G0300	MYB20	ACCCATCTCACCGTCCCAAA<br>TCTGCCTCCAGAGAGCTCCA	(Zhang et al., 2020)
PH01000030G0050	MYB63	AGGAGGACATGCGCCTCATC<br>TGAAGTTGCCGCGTTTGAGG	(Zhang et al., 2020)
PH01003093G0130	MYB85	AGGTCGACCCGCTGGTAAAG<br>TAGTCGAGCAGCCAGTTCGT	(Zhang et al., 2020)
PH01001753G0040	SND2	AGGGTGGCCATGGTGGTAAC<br>CCCTCCTGTGTGCACCTCAA	(Zhang et al., 2020)
PH01000107G0940	KNAT7	GCAGGACCTAACTGGTGCGA<br>TCCTGCCTGACCCTCTCCAT	(Zhang et al., 2020)
gi|242375393	CAC	AGTGAAACCGTTCCTTCCTCTGC<br>AGAACAATCTGCCAGTAACCTCA	(Fan et al., 2013)

References

Fan, C., Ma, J., Guo, Q., Li, X., Wang, H., Lu, M., 2013. Selection of Reference Genes for Quantitative Real-Time PCR in Bamboo (*Phyllostachys edulis*). PLOS ONE 8, e56573.

Hou, D., Lu, H., Zhao, Z., Pei, J., Yang, H., Wu, A., Yu, X., Lin, X., 2022. Integrative transcriptomic and metabolomic data provide insights into gene networks associated with lignification in postharvest Lei bamboo shoots under low temperature. Food Chemistry 368, 130822.

Li, C., Suo, J., Xuan, L., Ding, M., Zhang, H., Song, L., Ying, Y., 2019. Bamboo shoot-lignification delay by melatonin during low temperature storage. Postharvest Biology and Technology 156.

Zhang, H., Ying, Y., Wang, J., Zhao, X, Zeng, W., Beahan, C., He, J., Chen, X., Bacic, A., Song, L., Wu, A., 2018. Transcriptome analysis provides insights into xylogenesis formation in Moso bamboo (*Phyllostachys edulis*) shoot. Scientific Reports 8, 3951.

Zhang, Z., Li, C., Zhang, H., Ying, Y., Hu, Y., Song, L., 2020. Comparative Analysis of the Lignification Process of Two Bamboo Shoots Stored at Room Temperature. Plants 9.
